# Intraosseous papillary intralymphatic angioendothelioma (PILA): one new case and review of the literature

**DOI:** 10.1186/s13569-018-0087-9

**Published:** 2018-01-30

**Authors:** Marco Gambarotti, Alberto Righi, Marta Sbaraglia, Giuseppe Bianchi, Piero Picci, Daniel Vanel, Angelo Paolo Dei Tos

**Affiliations:** 1 Department of Pathology, Rizzoli Institute, Bologna, Italy; 2 Department of Pathology, Treviso Regional Hospital, Treviso, Italy; 3 Orthopaedic Oncology Department, Rizzoli Institute, Bologna, Italy; 4 University of Padua School of Medicine, Padua, Italy; 5 Istituto Ortopedico Rizzoli, Via di Barbiano 1/10, 40136 Bologna, Italy

**Keywords:** Papillary intralymphatic angioendothelioma, Dabska tumor, Bone

## Abstract

**Background:**

Papillary intralymphatic angioendothelioma (PILA) is a locally aggressive, rarely metastasizing vascular tumor, generally occurring in the soft tissues, with less than 40 cases described in the literature and only three cases reported in bone.

**Case presentation:**

We describe the case of a 51-year-old male with an intraosseous PILA of the proximal edge of his left clavicle and two other lesions evident on imaging. The patient was treated with marginal resection of the clavicle lesion but was lost to follow-up 1 month after surgery.

**Conclusions:**

PILA can also occur in bone, albeit very rarely, and has to be considered in the differential diagnosis of vascular bone tumors.

## Background

Papillary intralymphatic angioendothelioma (PILA) is a rare vascular tumor defined in the latest edition of the WHO tumor classification [1] as a “rarely metastasizing lymphatic vascular neoplasm”. It was initially considered a malignant tumor due to the fact that two patients had lymph node metastases [2]. The tumor was subsequently renamed PILA in 1998 by Fanburg-Smith et al. [3], considering its borderline behavior and prominent lymphatic phenotype. To the best of our knowledge, less than 40 cases of PILA have been described in the literature [2–17]. The majority of these cases occur in soft tissues, with only three cases reported in bone [2, 13, 15]. Due to its rarity, multifocality and morphological features, the diagnosis of PILA often represents a challenge for the pathologist.

The aim of the present study is to report a new case of PILA occurring in bone, describing the clinical, radiological, and histological features.

## Case presentation

Only one patient with intraosseous PILA was referred to and treated at our center between 1901 and 2016. This patient was a 51-year-old male with a painful lesion of the proximal edge of his left clavicle, which had been present for 2 months.

**Fig. 1:**
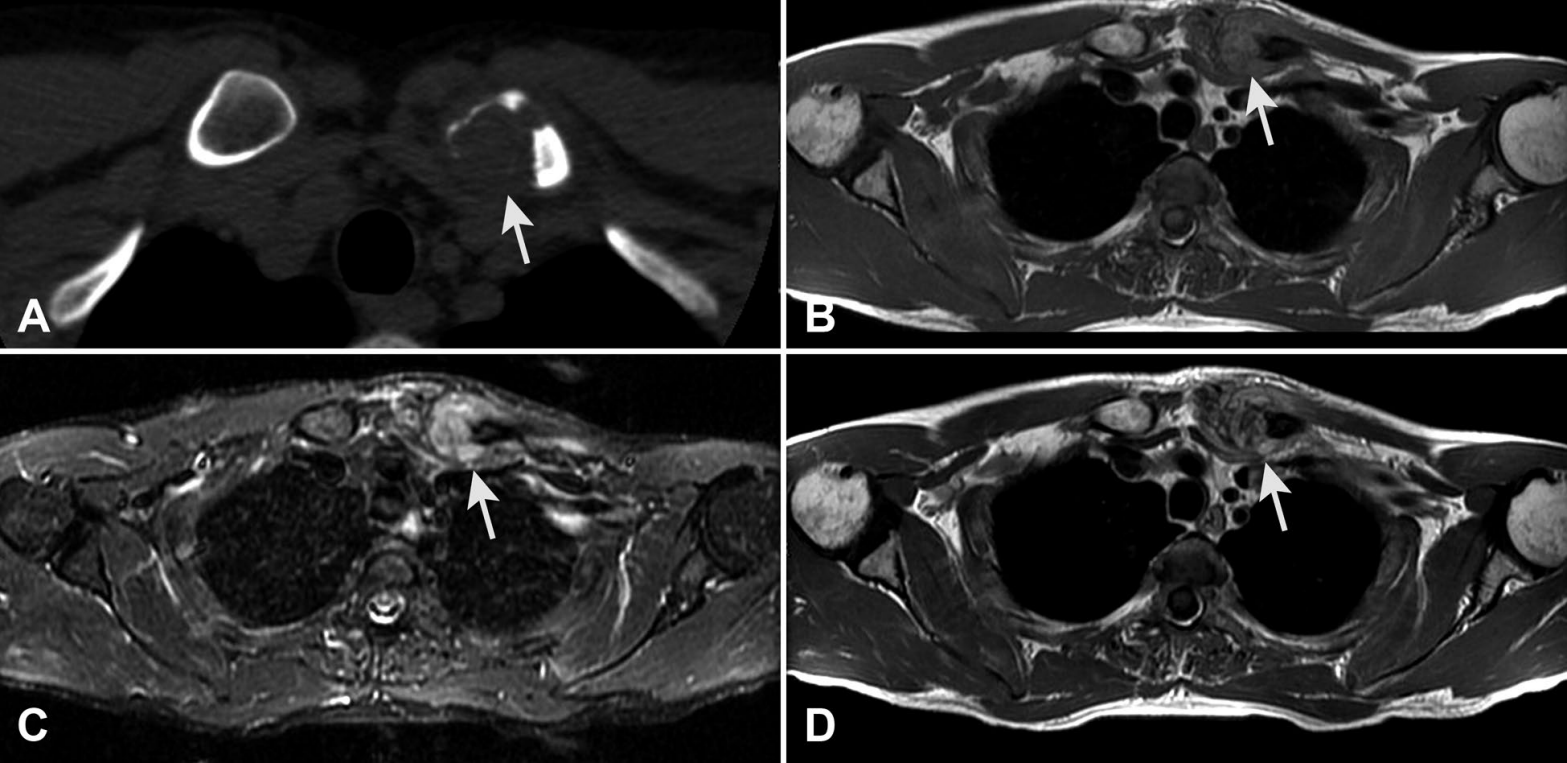
On CT **A** the lesion (see arrows) involves the medial end of the clavicle and is purely lytic; the cortex is destroyed and soft tissues are invaded. On axial MRI the signal is isointense on T1-weighted (**B**), and heterogeneously hyperintense on fat-saturated T2-weighted (**C**) and after contrast medium injection (**D**)

Imaging studies (Fig. 1A) revealed a lytic lesion in the medial end of the left clavicle with cortical destruction and soft tissue invasion. The signal was isointense on T1-weighted (Fig. 1B) and heterogeneously hyperintense on fat-saturated T2-weighted MRI (Fig. 1C) and after contrast medium injection (Fig. 1D).

Two other lesions were radiologically evident in the right clavicle and left distal femur.

The patient underwent a needle biopsy. Histologically, a vascular lesion was evident, with hypercellular areas and the neoplastic cells had a focal epithelioid appearance. A final diagnosis of low-grade hemangioendothelioma was made.

**Fig. 2:**
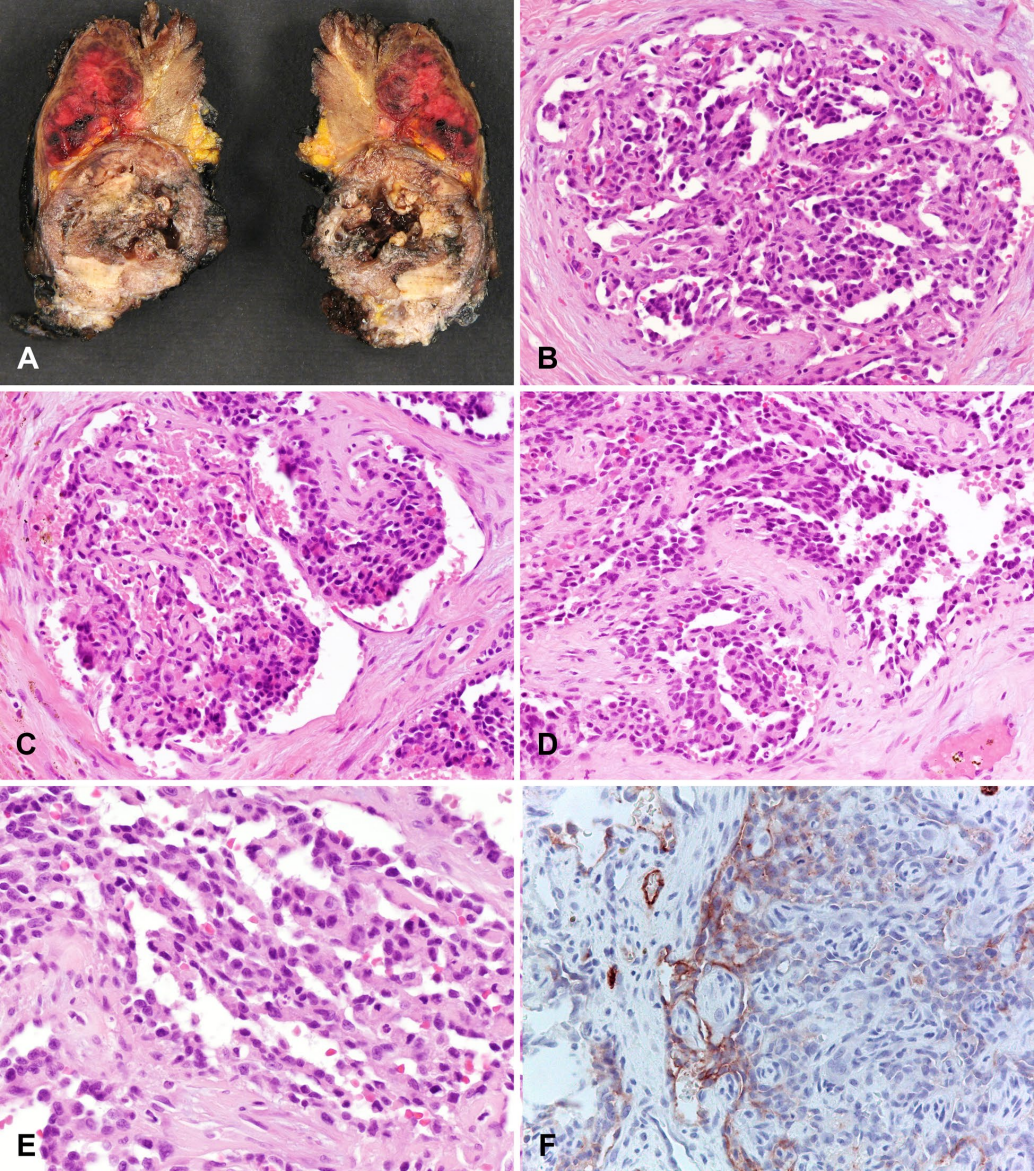
Gross features **A** an 8 cm. Lesion of the left clavicle, with soft tissue invasion. Histological features of the lesion, composed of glomerulus-like structures (**B**), with papillary structures projecting into the lumen, covered by plump “hobnail” endothelial cells (**C**–**E**). Immunohistochemical positivity for podoplanin (D2-40) in the neoplastic cells (**F**)

Partial resection of the clavicle was performed. Grossly, a gray-reddish lesion was present in the clavicle, with infiltration of the soft tissues (Fig. 2A). The tumor measured 8 × 4 × 3 cm. Histologically, the tumor was composed of multiple vascular channels, with some areas appearing as glomerulus-like structures (Fig. 2B), with papillary projections into the lumen (Fig. 2C–E). Papillae were covered by plump endothelial cells, some with a hobnail appearance. Immunohistochemically, the neoplastic cells were positive for podoplanin (D2-40), a lymphatic vessel marker (Fig. 2F), and for endothelial markers (CD31 and ERG). Surgical margins were focally marginal.

The patient was lost to follow-up 1 month after surgery.

## Discussion and conclusions

PILA is a rarely metastasizing lymphatic vascular neoplasm first described in 1969 by Dabska et al. [4]. To date less than 40 cases have been described in the literature [2–17].

Clinically, this extremely rare tumor more commonly occurs in infants, children and young adults [2, 13, 15], although it can also occur in the elderly [15] without sex predilection [15]. It is generally localized in the dermis and subcutaneous tissue of the extremities [3] and less commonly in the trunk, head and neck [2, 13]. Few cases have been reported in deeper locations, including the spleen [16], tongue [14], and testis [11, 17].

Only three cases have been reported in bone [2, 13, 15], as summarized in Table 1. The case reported by Li et al. [15] was characterized by multiple bone lesions in the facial bones. Our case was also characterized by multifocality on MR imaging. In the case described by McCarthy et al. [2], radiographs revealed periosteal reaction on the medial aspect of the distal femoral metaphysis and faint intraosseous radiolucency in the epiphysis. Almost identical features were described by Nakayama et al. [13]. In the case described by Bin et al. [15], CT-scan showed multifocal osteolytic lesions with soft tissue mass. On MRI the lesion showed low intensity on T1-weighted images and high intensity on T2-weighted images [2, 13].

**Table 1:** Clinicopathological features of papillary intralymphatic angioendotheliomas reported in literature

No	Age	Sex	Site	Treatment	Follow-up (months)	References
1	45	F	Distal femur (epiphysis)	Complete curettage	NED 12	[2]
2	39	F	Distal femur (metaphysis)	Curettage and wide re-excision	NED 500	[13]
3	1	M	Facial bones (multiple lesions)	Complete curettage	NED 24	[15]
4	51	M	Left clavicle (multiple lesions)	Marginal resection	Lost to follow-up	Current case

The pain and radiological features of the tumor are consistent with a well-circumscribed radiolucent lesion, generally with sclerotic margins on CT-scan, and are suggestive of osteoid osteoma [2, 13]. Langerhans cell histiocytosis also has to be considered in the differential diagnosis [15].

Histologically, PILA is composed of a proliferation of spindle- to polygonal-shaped, slightly atypical cells forming numerous interconnecting capillary and cavernous vascular cavities [2, 13, 15], with papillary projections into the lumen. The papillae consist of a fibrovascular core covered by slightly atypical plump cuboidal endothelial cells, with a hobnail or “match-head” appearance [2, 13, 15]. Solid areas and glomerulus-like structures may be present [15]. Mitoses are rare and necrosis is absent [15]. Immunohistochemically, the expression of podoplanin (D2-40) is consistent with its lymphatic phenotype [1].

Two vascular lesions, retiform hemangioendothelioma and papillary endothelial hyperplasia (Masson’s hemangioma), may be confused with PILA due to the presence of “hobnail” cells and papillary projections [2, 15]. However, the former is a locally aggressive, rarely metastasizing vascular tumor characterized by distinctive branching of arborizing blood vessels, imparting a pattern reminiscent of rete testis [2, 15]. The latter is a reactive endothelial proliferation with vascular thrombosis, characterized by papillary projections with hyaline or fibrin cores associated with thrombotic material, and often free-floating in the vascular lumens [2, 15].

“Hobnail” cells and papillary projections associated with podoplanin immunohistochemical expression are also helpful in distinguishing PILA from epithelioid hemangioma and lymphangioma-like Kaposi sarcoma [15].

PILA can be locally invasive with the potential to metastasize. Lymph node metastases have been reported [7] and a case of angiosarcoma arising within a PILA has been described [9]. In 2000 Dabska performed a 30-year review of the six patients originally reported in 1969: one patient died of widespread pulmonary metastases [18]. Accordingly, long-term follow-up should be performed in soft tissue PILA, although more recent series demonstrate no local recurrences or metastases [3] and an excellent prognosis with complete excision [2].

Due to the low numbers of intraosseous PILA reported in the literature, the difference between intraosseous and soft tissue PILA in tumor prognosis is unknown; similarly, no consensus has been reached as to the proper treatment of intraosseous PILA [13], although complete curettage seems sufficient to avoid recurrences [2].

In conclusion, we describe a new case of PILA of bone, with multifocal presentation on imaging. This very rare vascular tumor generally occurs in soft tissues, but can also be present in bone, thus extending the spectrum of vascular bone tumors.
